# Early Sacral Neuromodulation: A Promising Opportunity or an Overload for Patients with a Recent Spinal Cord Injury? A Cross-Sectional Study

**DOI:** 10.3390/jcm14031031

**Published:** 2025-02-06

**Authors:** Sophina Bauer, Lukas Grassner, Doris Maier, Ludwig Aigner, Lukas Lusuardi, Julia Peters, Orpheus Mach, Karin Roider, Evelyn Beyerer, Michael Kleindorfer, Andreas Wolff, Iris Leister, Elena E. Keller

**Affiliations:** 1 Institute of Molecular Regenerative Medicine, Paracelsus Medical University, 5020 Salzburg, Austria; sophina.bauer@gmail.com (S.B.);; 2 Department of Urology and Andrology, Landeskrankenhaus—University Clinic, 5020 Salzburg, Austria; 3 Spinal Cord Injury Center, BG Trauma Center Murnau, 82418 Murnau, Germany; 4 ParaMove, SCI Research Unit, BG Trauma Center Murnau, 82418 Murnau, Germany; 5 Department of Neurosurgery, Christian Doppler Clinic, Paracelsus Medical University, 5020 Salzburg, Austria; 6 Department of Neuro-Urology, BG Trauma Center Murnau, 82418 Murnau, Germany

**Keywords:** early sacral neuromodulation, spinal cord injury, neurogenic lower urinary tract dysfunction, quality of life

## Abstract

**Background:** A solid rationale exists for early sacral neuromodulation in the form of causal therapy that improves neurogenic lower urinary tract dysfunction after complete spinal cord injury. However, the short and early time frame for minimally invasive therapy poses a series of ethical and medical issues, which has impeded clinical realisation thus far. **Objectives**: We performed a cross-sectional study on patients with chronic spinal cord injury to learn about patients’ attitudes towards early treatment to prepare for large randomised controlled trials. **Methods:** A cohort of patients (n = 86, mixed genders) with spinal cord injury over two years was analysed. Their lower urinary tract-related quality of life was assessed using the Qualiveen-30 tool. The extent of neurogenic lower urinary tract dysfunction, patients’ awareness of it, and their attitude towards early sacral neuromodulation were explored with a specific questionnaire. **Results**: A total of 61.9% (n = 52) of patients declared that, in retrospect, they would have agreed to early treatment prior to the emergence of their autonomic dysfunction. Of these patients, 51.8% (n = 29) would have also consented to early sacral neuromodulation. Quality of life had no impact on their decision. More than half of the patients (n = 49, 57.0%) stated they had not grasped the momentous nature of neurogenic lower urinary tract dysfunction when being informed about it. This finding was subsequently correlated with a decreased lower urinary tract-related quality of life. **Conclusion**: Patients with neurogenic lower urinary tract dysfunction are likely to agree to an early therapeutic approach. Clinical implementation requires knowledge and acceptance of the procedure on the part of patients and their caregivers.

## 1. Introduction

Spinal cord injury (SCI) is a devastating condition and a life-changing event for patients. Besides motor and sensory deficits, which are the most obvious consequences of a damaged spinal cord, patients experience various autonomic dysfunctions, loss of sexual function, and, most importantly, dysfunction of the bowel and the bladder. Lower urinary tract (LUT) function is controlled by a complex neuronal network that includes peripheral nerves, the spinal cord, and higher brain centres. Depending on the level and severity of injury, SCI leads to distinct types of LUT malfunction. After a spinal lesion above the sacral micturition centre (S3), up to 95% of patients develop a neurogenic LUT dysfunction (nLUTD), with the neurogenic overactivity of the detrusor and, frequently, detrusor sphincter dyssynergia (DSD). These changes do not usually occur in the acute phase after SCI but typically develop over the first few months after the injury. The potential to develop DSD is largely not considered during the acute phase by the treating physician as well as the patient. DSD may ultimately lead to high pressure in the bladder, post-void residual volume, urinary tract infection (UTI), and incontinence. In the long term, the upper urinary tract is at high risk of deterioration, which, if untreated, may extend to complete kidney failure and the requirement for dialysis. Indeed, until the 1970s, one of the main causes of death after SCI was kidney failure [1].

Once developed, DSD and other forms of nLUTD may cause irreversible damage to bladder function and the bladder wall [2]. The currently established therapies focus on symptomatic treatment; neuro-urological care from an early stage with antimuscarinic therapy, botulinum toxin, and clean intermittent self-catheterisation (CISC) can prevent or lower chronic high pressure in the bladder, reduce UTIs, restore continence, and maintain kidney function for several years. However, we still lack a clinically established causal therapy that could prevent the development of DSD or preserve bladder function.

Sacral neuromodulation (SNM) proved to be a reasonable option to modulate bladder function, mainly for non-neurogenic bladder dysfunction. SNM is recommended by the European Association of Urology (EAU) as a third-line option for an overactive bladder along with botulinum toxin [3]. Even for nLUTD, SNM is an established procedure in patients with chronic incomplete SCI, Fowler’s syndrome, and other indications [4]. Sievert et al. were the first to report on the promising results of early SNM after complete SCI. After the early implantation of SNM in the acute shock phase after SCI, the patients remained with an acontractile detrusor, instead of developing the typical pattern of nLUTD with DSD [5]. Preclinical research confirmed these results in a minipig model. The authors registered a significant effect with less frequent development of DSD and better functional outcomes during a four-month follow-up period [6]. It seems that the stimulation of afferent neurons from the earliest possible time point after the occurrence of the spinal lesion may prevent the development of dyssynergia changes. After the manifestation of DSD in complete SCI, SNM has no relevant effect on nLUTD and is, therefore, not a suitable therapy [4]. This makes the time point of SNM immensely important and indicates that early SNM is a possible game changer in the search for causal therapy after complete SCI. However, early SNM is currently not a part of routine clinical practice after SCI.

In the acute and post-acute phases after SCI, SNM poses a series of ethical and medical issues, as this is the most sensitive period for the patient, including the following: the neurological scope is not clear and the patient might still require further surgery to stabilise the spinal cord. Neither the patient nor their professional caregivers (neurologists and neuro-urologists) are aware of the extent to which the LUT will function in the future. Addressing patients with no existing LUT symptoms in an early stage, convincing them of the necessity of surgery, albeit minimally invasive, and starting SNM therapy to maintain certain functionality of the LUT at a much later stage during recovery are demanding measures and require a sensitive approach.

To provide these patients with better counselling and thus facilitate their decision in favour of or against early SNM therapy, we conducted a cross-sectional quality-of-life (QOL) study. The main purpose was to understand how chronic SCI patients with persistent nLUTD would have decided about early, even invasive, therapy from the standpoint of their current knowledge about their dysfunction.

## 2. Materials and Methods

A cross-sectional study with a single cohort was designed as a descriptive and explorative survey to learn about patients with a chronic SCI, the extent of nLUTD and patients’ awareness of the same after two years, their LUT-related QOL, and their attitude towards early SNM.

One hundred patients were considered a representative sample size. The questionnaires were sent to the patients and the minimum response rate, set to 80%, was achieved. Inclusion criteria were written informed consent, knowledge of the German language, full legal competence, age over 18 years, and an SCI that had caused para- or tetraplegia more than two years ago.

We excluded patients with a pre-existing neurological disease unrelated to the SCI and those with congenital conditions (such as spina bifida, cerebral palsy, etc.) or progressive disease in the spinal cord (such as multiple sclerosis).

### 2.1. Study Design

All participants of the present study were part of the prospective, longitudinal, and population-based European Multicentre Study on Spinal Cord Injury (EMSCI) and were treated at a centre specialised in the management of SCI. The survey was carried out according to the Declaration of Helsinki and approved by the ethics committee of the Bavarian Medical Association (approval nr. 18065). Written informed consent was obtained from all participants.

**Figure 1:**
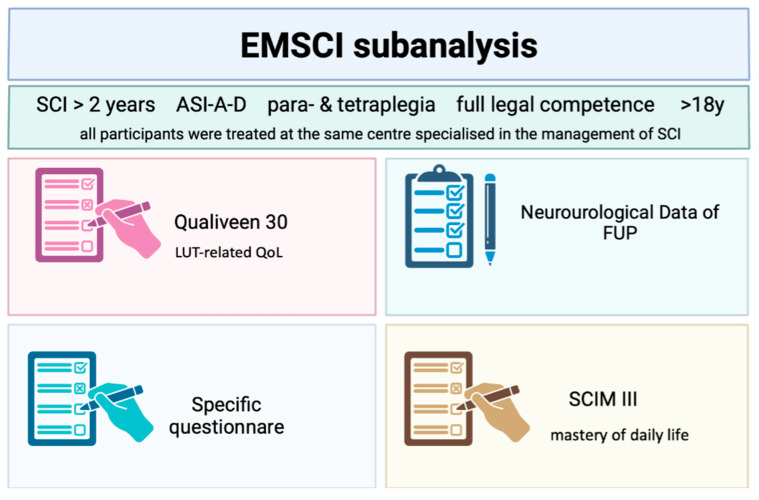
Overview of study design and questionnaires (created with Biorender^®^).

The patients were contacted by telephone or addressed personally during an outpatient visit. Patients who consented received a written informed consent form and three questionnaires. The envelopes with the returned questionnaires were rendered anonymous and, together with the anonymised and relevant neuro-urological clinical records, handed over to a blinded team that had not been previously involved in the patients’ care (see Figure 1). The blinded team evaluated the data and performed the statistical analysis. The clinical records included the SCI report with data about the cause of SCI, complications, surgeries, medication, the initial American Spinal Injury Association Impairment Scale (AIS) score, neuro-urological data on the method of bladder emptying, UTIs, sexual function, bowel management, reflexes, secondary urological diagnoses and surgical procedures (such as transurethral resection of the prostate for prostate enlargement), ultrasonography of the kidneys, and video-urodynamic (VUD) parameters at every clinical visit.

The time points of the first and the most recent urological examinations were taken for the analysis and comparison of parameters, including (video-) urodynamic parameters and diagnosis.

### 2.2. Questionnaires

Qualiveen-30, an internationally established tool for recording a patient’s LUT-related QOL in a sociodemographic context, was chosen and is graded between zero (best possible LUT-related QOL) and four (worst LUT-related QOL). To evaluate the mastery of daily life for patients with an SCI, the SCIM III was selected. A high independence in activities of daily life scores towards 100. The third questionnaire was specifically designed in order to obtain the patient’s retrospective view of their nLUTD. The questionnaire included clarification of the condition by the urologist, the patient’s realisation of the momentousness of their nLUTD, and particularly the patient’s willingness to undergo SNM shortly after the early SCI. Finally, the participants were asked what type of therapy they would approve of today to improve their bladder function.

### 2.3. Statistics

All data of continuous variables were checked for normal distribution (test of normality: Kolmogorov–Smirnov with Lilliefors significance correction, type I error = 10%). Variables with normally distributed data were compared between lesion-specific subgroups (spinal C2-L1 vs. sacral L2-S5) by using the *t*-test for independent samples (test for variance homogeneity: Levene’s test, type I error = 5%). Otherwise, and for variables measured on ordinal scales, we used the exact Mann–Whitney U test. Dichotomous variables were compared with Fisher’s exact test and all other categorical variables with the exact chi-square test.

Intra-individual visit comparisons of continuous variables with normally distributed data were performed with the paired *t*-test. Otherwise, and for variables measured on ordinal scales, we used the exact Wilcoxon test. Categorical variables were compared with the exact McNemar test.

Correlations were analysed as follows:

- Dichotomous variable vs. dichotomous variable: phi coefficient.
- Dichotomous variable vs. categorical (non-dichotomous) variable: Cramer’s V.
- Dichotomous variable vs. ordinal variable or continuous (not normally distributed) variable: point-biserial Spearman’s rank correlation coefficient.
- Continuous variable vs. ordinal variable or continuous (not normally distributed) variable: Spearman’s rank correlation coefficient.

Relationships between continuous (not normally distributed) and categorical (non-dichotomous) variables were investigated using the eta coefficient and the Kruskal–Wallis one-way analysis of variance.

Since the type I error was not adjusted for multiple testing, the results of inferential statistics are only descriptive and the use of the term “significant” in the description of the study results always reflects only a local *p* < 0.05 but no error probability below 5%.

Statistical analysis was performed using the open-source R statistical software package, version 3.6.1 (The R Foundation for Statistical Computing, Vienna, Austria).

We performed a sub-analysis of the success of video-urodynamic (VUD) investigations based on the method proposed by Pannek et al. [7]. The latter authors divided their urodynamic results into successful and unsuccessful categories using the following criteria for success: capacity over 360 mL, detrusor pressure below 40 cm H_2_O, and absence of incontinence and autonomic dysreflexia. If only one of these parameters did not achieve said value, the patients’ urodynamics were deemed unsuccessful. In our analysis, we had to exclude autonomic dysreflexia, as no continuous records were available on the subject, and we only performed this sub-analysis later to complement our results.

## 3. Results

### 3.1. Participants

Of 181 invited patients, 100 consented to participate in this study. We excluded 14 patients due to incomplete data. Finally, 86 valid cases were used for the analysis.

All patients were initially treated and underwent all follow-up measurements at the Murnau Trauma Hospital in Germany. Eighty-six per cent (n = 74) of the participants were male, and the patients’ mean age at the time of the survey was 47.24 years (range of 34–74 years). Nearly all patients (n = 83, 96.5%) were paraplegic, and 37.0% (n = 43) had a complete SCI for more than two years. In total, 69 patients (81.2%) had a traumatic SCI, of which 48 (57.8%) had multiple traumata, 68 (81.0%) had a fracture in their vertebra/spine, and 64 (77.1%) required stabilising surgery in the spine.

Seventy-seven patients (89.5%) had their injury at a level associated with a strong likelihood of DSD (spinal lesion), while a mere nine patients had a low lesion below the sacral micturition centre with impaired or disabled emptying of the bladder. Further data about patients are provided in Table 1.

**Figure 2:**
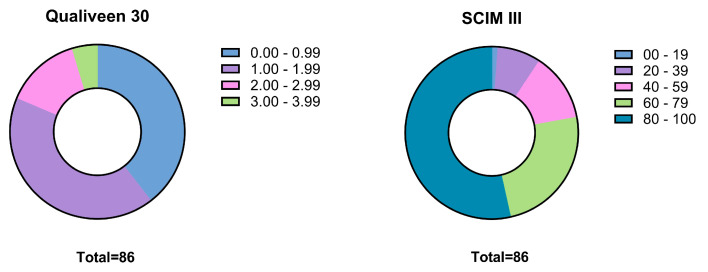
Qualiveen-30 and SCIM III patient distribution.

The participants had a mean score of 1.28 on the Qualiveen-30 (0–4) questionnaire and 70.02 on the SCIM III (0–100) questionnaire (Figure 2).

### 3.2. Neuro-Urological Follow-Up

Patients had an indwelling transurethral catheter for a mean period of 4.82 weeks (SD 3.0; range: 0.3–13.0) after the injury. At the time of the first urodynamic evaluation, more than half of the patients (n = 45; 60.0%) performed CISC and 16 had a suprapubic catheter (21.3%). At the time of the last VUD investigation, 50 participants (72.5%) used CISC on a regular basis while 13 (19.1%) remained with a suprapubic catheter (Table 1).

At the last neuro-urological investigation, 32.8% (n = 19) of patients had urinary incontinence (Table 1). Compared with their first visit, 22.5% of patients experienced a worsening of their incontinence, 7.5% improved, and the vast majority (70.0%) experienced no change.

The predominant pattern of nLUTD at the last visit was neurogenic detrusor overactivity with DSD (n = 31, 44.3%). This coincided with the fact that the majority of patients had a spinal lesion above the micturition centre (n = 77, 89.5%). A detailed list of neurological diagnoses and current therapies is provided in Table 1.

Of the male patients who received follow-up assessment in regard to their sexual function, 26 (n = 26/46, 56.5%) said at their last visit that they had erections and 12 were able to achieve ejaculation (n = 12/44, 27.3%) and an orgasm (n = 12/46, 26.1%).

Nearly all patients (n = 45/48, 93.8%) required treatment for their bowel evacuation. The aids used were mainly suppositories (n = 16, 33.3%), laxatives (n = 11, 22.9%), and digital manoeuvres (n = 13, 27.1%) (Table 1).

The mean period between the first and the last VUD evaluation was 2.16 years. Detailed information about the urodynamic investigations is provided in Table 2.

### 3.3. Specific Questionnaire

**Figure 3:**
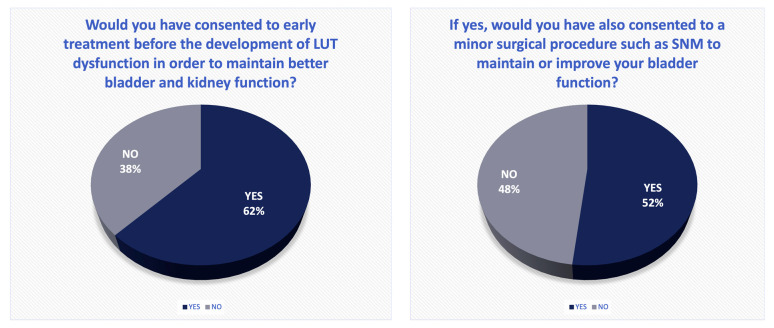
Legend: The results of the specific questionnaire concerning early treatment after SCI. A total of 62% of the participants would have consented to an early treatment even before the development of LUT dysfunction. Of these patients, another 52% would have also consented to a minimally invasive procedure such as sacral neuromodulation.

The majority of the patients (n = 67, 87.0%) first experienced neurogenic lower urinary tract symptoms (LUTSs) six months after the SCI. Six patients (7.8%) reported no LUTSs whatsoever, and 72.4% (n = 55) were directly informed about upcoming changes in bladder function during their hospital stay after the SCI. From the patients’ perspectives, 17.1% (13 patients) received sufficient information to comprehend the magnitude of their bladder dysfunction during their rehabilitation stay. Furthermore, 9.2% (seven patients) stated that they were initially informed about bladder dysfunction at the onset of symptoms. One patient (1.3%) did not remember the information at all. More than half of the participants (n = 49, 57.0%) stated they had not realised the momentous nature of nLUTD when they were informed about it, while 61.9% (n = 52) said they would have consented to early therapy prior to the emergence of their nLUTD in order to maintain better bladder and kidney function (Figure 3). Of these patients, 51.8% (n = 29) would have also consented to undergo SNM. Regarding the question as to which therapy they would accept today in their current chronic situation, 26.5% (n = 22) said they were open to new approaches in the course of clinical trials. An equivalent number of patients (n = 13, 15.7%) voted for SNM, botulinum toxin, and drug therapy only as further options. Eighteen patients (21.7%) would not consent to any further therapy.

### 3.4. Correlation Analysis

We correlated the Qualiveen-30 scores with the neuro-urological data of the patients in order to determine relevant characteristics with regard to QOL.

**Figure 4:**
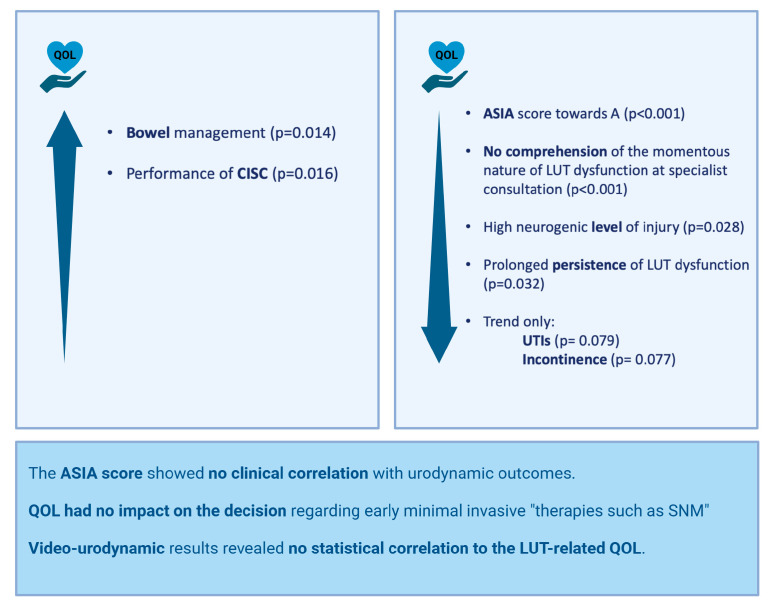
Correlation analysis of quality of life (created with Biorender^®^).

Poor QOL was correlated with a high neurogenic level of injury, an American Spinal Injury Association (ASIA) Impairment Scale (AIS) score towards A, and prolonged persistence of nLUTD. Patients who performed CISC had a higher QOL score compared with indwelling catheter drainage. Preserved bowel function without the requirement for bowel management procedures was correlated with high QOL (Figure 4).

A significant negative impact on QOL was observed in those patients who did not comprehend the momentous nature of nLUTD when the urologist informed them about their bladder situation. QOL had no impact on the decision regarding early minimally invasive therapies such as SNM.

We did not register any impact of VUD outcomes on patients’ QOL. A sub-analysis of defined success in the urodynamic investigation (adapted from protocols of Pannek et al. [7]) again revealed no statistically significant correlation between the results of the VUD investigation and the Qualiveen-30 questionnaire. The AIS score showed no correlation with urodynamic outcomes.

## 4. Discussion

### 4.1. Patient Population

All patients who experienced SCI were treated at the same centre. Thus, inter-hospital differences in patient management and performance were ruled out. Mainly patients with paraplegia consented to participate in the survey but those with a lesion above the sacral micturition centre (named the spinal group) also participated. This explains the high rates of CISC and the main neuro-urological diagnosis of neurogenic detrusor overactivity with DSD. Due to the small number of patients in the sacral lesion group, a comparison between the sacral and the spinal group was statistically not feasible.

The patients had a high LUT-related QOL score on the Qualiveen-30 questionnaire, a relatively low detrusor pressure even over a longer period, and a low requirement for botulinum toxin or more invasive therapies. These favourable long-term results, along with high scores on the SCIM III, can be explained by regular controls and high compliance with the therapy. However, it should be noted that not all patients have access to continued neuro-urological care of high quality during follow-up. This might have signified a bias in the present survey, as the LUT-related QOL registered here may not reflect the average population.

### 4.2. Quality of Life

Impaired bladder or bowel function is known to reduce QOL in patients with an SCI [7,8,9]. Overall, our patients had a relatively high LUT-related QOL. Preserved bowel function without the requirement for bowel management procedures and CISC were significantly correlated with high QOL. Recurrent UTIs and urinary incontinence tended to have a negative impact on QOL, without achieving significance. This may be due to the retrospective nature of the analysis; the above-mentioned aspects were not recorded in detail.

The present results reflect the current published data, although UTIs and urinary incontinence only had a general impact on QOL in our survey.

The results of VUD studies showed no correlation with QOL. We applied an adapted version of Pannek et al.’s method [7] to detect an underlying association between urodynamic parameters and QOL in our primarily non-correlated results. Although two-thirds of the participants had a successful urodynamic investigation, we found no statistically relevant correlation with the total Qualiveen-30 score. Therefore, in view of the exact calculation of a correlation between QOL and urodynamics provided by the above-mentioned authors, we suggest that future investigations adhere to the original protocol of Pannek et al. from the outset.

The specific questionnaire employed in this study provided important information about patients’ subjective impression of their early neuro-urological care. Although about 90% of the patients were informed early about their SCI-related bladder changes at a specialised neuro-urology centre, less than half comprehended the momentous nature of these changes, which had a significant negative impact on their QOL. This underlines the importance of neuro-urologists providing regular and extensive counselling to patients with SCI.

Of patients who live with an nLUTD after SCI, more than 60% said they would opt for early treatment even before the onset of symptoms. We did not inform the patients in detail about SNM in the context of this survey, yet a further 50% of patients claimed they would have consented to this minimally invasive procedure. To rule out general dissatisfaction as a motivation for retrospective consent to early treatment, we tested the relation between the bladder-related QOL and each patient’s decision but registered no impact of QOL on the decision for or against early treatment.

No association was registered between sexual function and QOL. However, this must be viewed with caution because not all patients were asked about their sexual lives during the follow-up period.

### 4.3. Sacral Neuromodulation

According to the European Association of Urology (EAU) Guidelines on Neuro-Urology, SNM is a reliable, effective, and strongly recommended treatment for non-neurogenic dysfunction of the LUT [3]. In recent decades, there has been growing evidence for SNM indications for nLUTD [4]. Recently a sham-controlled, double-blind, multicentre trial proved the safety and efficacy of SNM for nLUTD. Neurostimulators were implanted in patients with refractory nLUTD and stimulation was performed for a 2-month test phase. Nine patients with incomplete chronic SCI were included. In patients whose LUT function improved by over 50% from baseline, neurostimulation was randomly continued or discontinued for a further two months. In the patient cohort with continued stimulation, a success rate of 76% could be indicated. The SCI patients showed similar results compared with other nLUTD indications. As both early and late SNM in complete SCI are not part of the clinical routine today, none such patients were included in the survey [10]. In summary, SNM is a safe and effective option in incomplete SCI patients [3,4,10]. Concerning complete SCI, SNM could not improve nLUTD once the typical pathology manifested [11,12]. Sievert et al. suggested the provision of treatment in the acute phase after complete SCI and started SNM between 0.8 months and 4.5 months after the initial traumatic incident. Early SNM before the development of DSD showed promising results with low intravesical pressures and a remaining acontractile detrusor [5]. Preclinical studies have shown that SNM is an effective treatment for neurogenic dysfunction of the LUT. Our own preclinical work already proved the efficacy of eSNM in a standardised SCI minipig model [6,13]. After a complete thoracic SCI, the animals received eSNM. Urodynamics and functional tests were conducted at regular intervals during four months of follow-up post-SCI. eSNM-treated minipigs did not develop DSD and showed improved bladder function. Structural analysis of the bladder wall showed less fibrotic progression. Additionally, a recent study also showed the efficacy of eSNM after complete SCI in a rodent model. The treatment increased the bladder capacity significantly and decreased non-voiding contraction as well as the voiding efficacy compared with the untreated group [14].

In light of these data with preclinical confirmation of eSNM effectiveness and the first promising clinical data points, there is still a lack of any long-term data. We await the results of the announced American study by Redshaw et al. who published a protocol for a randomised clinical trial after acute SCI [15]. The authors intend to implant an SNM device within 12 weeks after SCI in 30 patients. The authors plan to continue the regular treatment regimen simultaneously and conduct follow-up assessments of the patients for one year. Although we found no data on the recruitment plan and clinical feasibility, the results of this study could shed light on the question of whether SNM can be part of the clinical routine. Until we have solid results on eSNM, this technique remains investigational [16]. Besides the proof of concept, another challenge that remains is the clinical implementation of eSNM. Our literature research could not provide specific information concerning the clinical challenges and implications for SNM in SCI, regardless of the time point of application.

However, timing might be a key factor in the treatment after complete SCI. Usually, SNM is a second- or third-line therapy for other LUTD indications. Thus, urologists have enough time to consider the pros and cons for each patient and establish the patient’s suitability for SNM. After an SCI, both patients and caregivers are confronted with a new and acute situation that involves several other difficulties and dysfunctions in addition to those in the urinary tract. Moreover, nLUTD and all its facets are usually manifested several months after SCI. At this time, when the patient becomes aware of the momentous nature of nLUTD, the time frame for SNM has passed. The intervention should ideally be performed during the acute spinal shock [5]. The main ethical question is as follows: is the treatment an overload for patients with a fresh SCI? Offering patients an invasive therapy even before the patients or caregivers are aware of the full extent of the neurological lesion is a debatable issue. Can we really predict how much of the individual LUT function can be salvaged with SNM? Or should we regard SNM as a means of preventing damage to the LUT, which we know develops in more than 90% of patients with an SCI?

Our data showed that the patients would retrospectively consent to early therapy, and some would even welcome new therapies. As a result of our recent experiences, we have learned that nLUTD is a key factor in SCI-related QOL. However, SNM is not fully understood yet [17]. What is known is that this therapy is minimally invasive, fully reversible, and can be turned off by the patient at any time. Therefore, we should be able to offer this therapy in widespread clinical trials to all patients who would be willing to try it. Nevertheless, a constructive dialogue between the caregiver and the patient is of utmost importance. The clinician must ensure the patient understands the highly probable condition of nLUTD and the possibilities and limitations of SNM. Furthermore, it is necessary to educate other involved medical caregivers, neurologists, neurosurgeons, nurses, and rehabilitation centres about the importance of early commencement of this therapy. An open discussion is required about the independence of nLUTD and the AIS score [18], and subsequently, it should be conveyed that the decision of whether a patient is eligible for early SNM is independent of injury severity by means of the AIS score. Further investigation of these considerations may permit clinical implementation of this therapy and the subsequent performance of large clinical trials to further prove the efficacy of early SNM in complete SCI.

## 5. Conclusions

In retrospect, our patients would have consented to early therapy such as SNM.

Early SNM could be a clinical game changer in SCI-related nLUTD, but only if patients and caregivers are well informed. Educating urologists, neurologists, neurosurgeons, and caregivers will permit the clinical implementation of standard operation procedures (SOPs) so that larger clinical trials can be performed to further prove the efficacy and clinical feasibility of the treatment.

Despite discussions about early SNM, what matters most to the patients is QOL. The latter could be improved by early and detailed clarification of autonomic malfunctions of the LUT after SCI by a specialist, a holistic approach to the treatment, early attention to bowel and sexual function, and CISC wherever feasible.

## Figures and Tables

**Table 1:** Patients’ data.

Time point of the last neuro-urological evaluation	
Sex (male n (%))	74 (86.0)
Age at the time of the questionnaire in years (median + IQR)	47.5 (±15.69)
Cause of SCI (traumatic n (%))	69 (81.2)
Neurogenic level of injury (paraplegia n (%))	83 (96.5)
Cervical n (%)	5 (6.2)
Thoracic n (%)	63 (77.8)
Lumbar n (%)	13 (16.0)
ASIA Impairment Scale (AIS, n, (%))	
A n (%)	34 (40.0)
B n (%)	14 (16.5)
C n (%)	10 (11.8)
D n (%)	27 (31.8)
Neuro-urological diagnosis n (%)	
Neurogenic detrusor overactivity (NDO) n (%)	12 (17.1)
NDO with DSD n (%)	31 (44.3)
Incontinence n (%)	19 (32.8)
Recurrent urinary tract infection n (%)	30 (46.2)
Anticholinergic therapy n (%)	24 (35.3)
Botulinum toxin therapy n (%)	12 (17.9)
Method of bladder emptying	
Clean intermittent self-catheterisation n (%)	50 (72.5)
Suprapubic catheter n (%)	13 (19.1)
Indwelling catheter n (%)	6 (9.0)
Aids required for bowel management n (%)	45 (93.8)
None	3 (6.3)
Laxatives	11 (22.9)
Suppositories	16 (33.3)
Digital manoeuvres	13 (27.1)
Irrigation	1 (2.1)
Combinations	4 (8.3)
Sexual function	
Erection in males possible n (%)	26 (56.6)
Ejaculation in males possible n (%)	12 (27.3)
Sensation of orgasm possible n (%)	12 (26.1)

**Table 2:** Video-urodynamic data.

Maximal bladder capacity (mL) (mean ± SD)	526.75 (±170.35)
Detrusor pressure (cm H_2_O) (mean ± SD)	28.22 (±24.06)
Bladder compliance (cm H_2_O/mL) (median, min.–max.)	42 (7–∞)
Detrusor sphincter dyssynergia n (%)	39 (60.0)
Incontinence observed in VUD n (%)	3 (6.4)
Detrusor contractility during voiding n (%)	11 (17.2)
Post-void residual volume (mL) (mean ± SD)	488.69 (±236.03)
Successful VUD n (%)	43 (62.3)

## Data Availability

The datasets used and/or analysed during the current study are available from the corresponding author upon reasonable request.

## Abbreviations

AIS	American Spinal Injury Association (ASIA) Impairment Scale
ASIA	American Spinal Injury Association
CISC	Clean intermittent self-catheterisation
DSD	Detrusor sphincter dyssynergia
EMSCI	European Multicentre Study on Spinal Cord Injury
EAU	European Association for Urology
LUT	Lower urinary tract
LUTD	Lower urinary tract dysfunction
LUTSs	Lower urinary tract symptoms
nLUTD	Neurogenic lower urinary tract dysfunction
NDO	Neurogenic detrusor overactivity
QOL	Quality of life
SCI	Spinal cord injury
SCIM	Spinal cord independence measure
SD	Standard deviation
SNM	Sacral neuromodulation
UTI	Urinary tract infection
VUD	Video-urodynamics
